# 8-*O*-Ethyl­yunaconitine from the roots of *Aconitum carmichaeli* Debx.

**DOI:** 10.1107/S1600536812026463

**Published:** 2012-06-30

**Authors:** San-Lin Wu, Fang Liu

**Affiliations:** a Department of Chemistry and Life Sciences, Leshan Teachers College, Leshan 614004, People’s Republic of China

## Abstract

The title compound [systematic name: (1α,3α,6α,8β,13β,14α,16β)-20-ethyl-8-eth­oxy-3,13-dihy­droxy-1,6,16-trimeth­oxy-4-(meth­oxy­meth­yl)aconitan-14-yl 4-meth­oxy­benzoate], C_35_H_51_NO_10_, was isolated from roots of *Aconitum carmichaeli* Debx., which is a typical C_19_-diterpenoid alkaloid. The mol­ecule has an aconitane carbon skeleton with four six-membered rings and two five-membered rings. The six-membered rings adopt chair conformations or boat conformations, while the five-membered rings have envelope conformations. Intra­molecular O—H⋯O and O—H⋯N hydrogen bonds help to stabilize the mol­ecular structure. Weak inter­molecular C—H⋯O inter­actions occur in the crystal structure.

## Related literature

For a related structure, see: Wang *et al.* (2009 ▶).
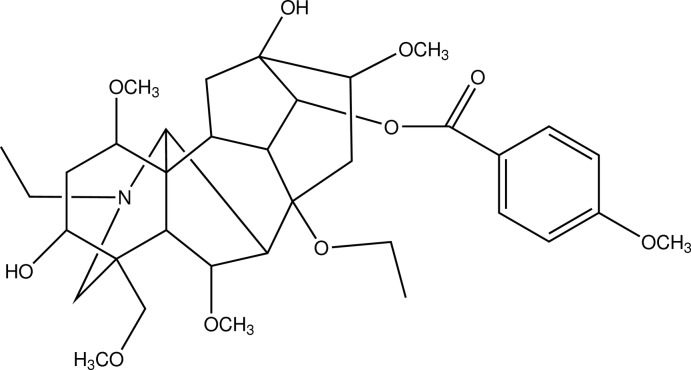


## Experimental

### 

#### Crystal data

C_35_H_51_NO_10_
*M* *_r_* = 645.77
Monoclinic, 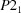

*a* = 10.0176 (4) Å
*b* = 11.7075 (5) Å
*c* = 14.3449 (5) Å
β = 92.528 (3)°
*V* = 1680.75 (11) Å^3^
*Z* = 2
Mo *K*α radiation
μ = 0.09 mm^−1^
*T* = 293 K
0.41 × 0.40 × 0.38 mm

#### Data collection

Oxford Diffraction Xcalibur Eos diffractometer
7371 measured reflections
3609 independent reflections
2756 reflections with *I* > 2σ(*I*)
*R*_int_ = 0.031

#### Refinement

*R*[*F* ^2^ > 2σ(*F* ^2^)] = 0.053
*wR*(*F* ^2^) = 0.131
*S* = 1.06
3609 reflections
438 parameters
1 restraint
H atoms treated by a mixture of independent and constrained refinement
Δρ_max_ = 0.27 e Å^−3^
Δρ_min_ = −0.17 e Å^−3^

### 

Data collection: *CrysAlis PRO* (Oxford Diffraction, 2009 ▶); cell refinement: *CrysAlis PRO*; data reduction: *CrysAlis PRO*; program(s) used to solve structure: *SHELXTL* (Sheldrick, 2008 ▶); program(s) used to refine structure: *SHELXTL*; molecular graphics: *SHELXTL*; software used to prepare material for publication: *SHELXTL*.

## Supplementary Material

Crystal structure: contains datablock(s) I, global. DOI: 10.1107/S1600536812026463/xu5540sup1.cif

Structure factors: contains datablock(s) I. DOI: 10.1107/S1600536812026463/xu5540Isup2.hkl

Additional supplementary materials:  crystallographic information; 3D view; checkCIF report

## Figures and Tables

**Table 1:** Hydrogen-bond geometry (Å, °)

*D*—H⋯*A*	*D*—H	H⋯*A*	*D*⋯*A*	*D*—H⋯*A*
O2—H2⋯O1	0.89 (6)	2.32 (6)	2.932 (4)	126 (5)
O2—H2⋯N1	0.89 (6)	2.21 (6)	2.845 (4)	128 (5)
O5—H5⋯O7	0.91 (5)	1.99 (6)	2.562 (5)	120 (4)
C35—H35*B*⋯O2^i^	0.96	2.56	3.245 (5)	129

Symmetry code: (i) 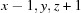.

**Fig. 1.**
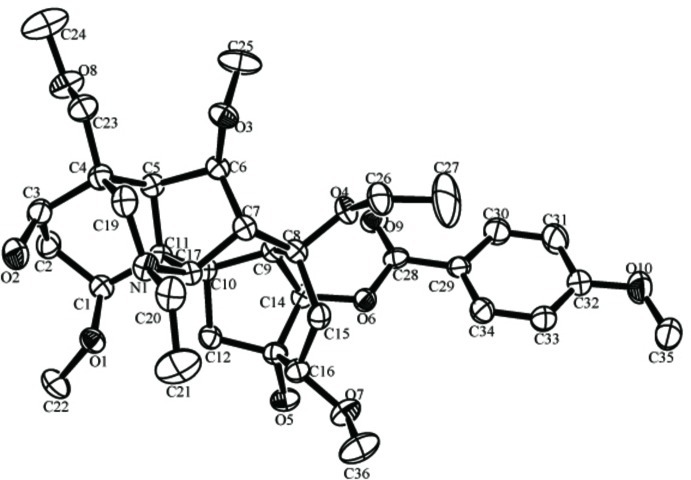
The molecular structure of the title compound with 30% probability displacement ellipsoids for non-H atoms.

**Fig. 2.**
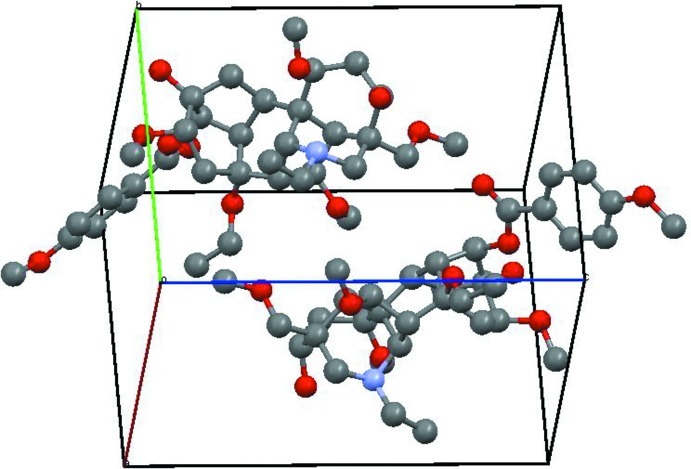
Molecular packing of the title compound.

## supplementary crystallographic information

### Comment

The title compound, 8-*O*-ethylyunaconitine, was previously isolated from *Aconitumcarmichaeli* Debx., and its structure was established from the NMR and MS data. In our recent investigation, it was isolation from the root of *Aconitum carmichaeli* Debx, collected in the E'mei Mountain, Sichuan Province of China in August 2010, and its crystal structure was determined (Wang *et al.*, 2009).

The molecular structure of the title compound is shown in Fig. 1. Six-membered rings A (C1/C2/C3/C4/C5/C11) and D (C8/C9/C14/C13/C16/C15) adopt boat conformations; six-membered ring B (C7/C8/C9/C10/C11/C17) adopts chair conformation; six-membered heterocyclic ring E (C4/C5/C11/C17/N1/C19) adopts the same chair conformation; the five-membered rings C (C9/C10/C12/C13/C14) and F (C5/C6/C7/C17/C11) display an envelope conformation, in which, the C14 and C11 act as the "envelope" respectively. The crystal structure contains intermolecular O—H···O and O—H···N hydrogen bonds. The intermolecular hydrogen bonds may be effective in the stabilization of the structure.

The absolute configuration of the title compound can not be confirmed by the present MoKa diffraction data. But it can be assumed to be the same as that reported for C_19_-diterpenoid alkaloids from the nature (Wang *et al.*, 2009).

### Experimental

Air-dried and powdered roots (600 g) were percolated with 0.1 *M* HCl (6 *L*). The obtained acid aqueous solution was basified with 10% aqueous NH_4_OH to pH 11 and then extracted with ethyl acetate (6 *L* × 3). Removal of the solvent under reduced pressure afforded the total crude alkaloids (5.2 g) as a yellowish amorphous powder, which was chromatographed over a silica gel column, eluting with cyclohexane-acetone (7:1→1:2) gradient system, to afford 8-*O*-ethylyunaconitine (256 mg). The crystals suitable for X-ray structure analysis was obtained by slow evaporation from an acetone solution at room temperature.

### Refinement

moiety Hydroxyl H atoms were located in a difference Fourier map and refined isotropically. Other H atoms were located geometrically with C—H = 0.93–0.98 Å, and refined using a riding model with *U*_iso_(H) =1.2*U*_eq_(C). The absolute configuration has not been determined for the structure.

### Crystal data

**Table:**

C_35_H_51_NO_10_	*F*(000) = 696
*M**_r_* = 645.77	*D*_x_ = 1.276 Mg m^−^^3^
Monoclinic, *P*2_1_	Mo *K*α radiation, λ = 0.7107 Å
*a* = 10.0176 (4) Å	Cell parameters from 2465 reflections
*b* = 11.7075 (5) Å	θ = 3.1–29.1°
*c* = 14.3449 (5) Å	µ = 0.09 mm^−^^1^
β = 92.528 (3)°	*T* = 293 K
*V* = 1680.75 (11) Å^3^	Block, colorless
*Z* = 2	0.41 × 0.40 × 0.38 mm

### Data collection

**Table:**

Oxford Diffraction Xcalibur Eos diffractometer	2756 reflections with *I* > 2σ(*I*)
Radiation source: Enhance (Mo) X-ray Source	*R*_int_ = 0.031
Graphite monochromator	θ_max_ = 26.4°, θ_min_ = 3.1°
Detector resolution: 16.0874 pixels mm^-1^	*h* = −6→12
ω scans	*k* = −13→14
7371 measured reflections	*l* = −17→17
3609 independent reflections	

### Refinement

**Table:**

Refinement on *F*^2^	Primary atom site location: structure-invariant direct methods
Least-squares matrix: full	Secondary atom site location: difference Fourier map
*R*[*F*^2^ > 2σ(*F*^2^)] = 0.053	Hydrogen site location: inferred from neighbouring sites
*wR*(*F*^2^) = 0.131	H atoms treated by a mixture of independent and constrained refinement
*S* = 1.06	*w* = 1/[σ^2^(*F*_o_^2^) + (0.0547*P*)^2^ + 0.155*P*] where *P* = (*F*_o_^2^ + 2*F*_c_^2^)/3
3609 reflections	(Δ/σ)_max_ < 0.001
438 parameters	Δρ_max_ = 0.27 e Å^−^^3^
1 restraint	Δρ_min_ = −0.17 e Å^−^^3^

### Special details

**Table:**

Geometry. All e.s.d.'s (except the e.s.d. in the dihedral angle between two l.s. planes) are estimated using the full covariance matrix. The cell e.s.d.'s are taken into account individually in the estimation of e.s.d.'s in distances, angles and torsion angles; correlations between e.s.d.'s in cell parameters are only used when they are defined by crystal symmetry. An approximate (isotropic) treatment of cell e.s.d.'s is used for estimating e.s.d.'s involving l.s. planes.
Refinement. Refinement of *F*^2^ against ALL reflections. The weighted *R*-factor *wR* and goodness of fit *S* are based on *F*^2^, conventional *R*-factors *R* are based on *F*, with *F* set to zero for negative *F*^2^. The threshold expression of *F*^2^ > σ(*F*^2^) is used only for calculating *R*-factors(gt) *etc*. and is not relevant to the choice of reflections for refinement. *R*-factors based on *F*^2^ are statistically about twice as large as those based on *F*, and *R*- factors based on ALL data will be even larger.

### Fractional atomic coordinates and isotropic or equivalent isotropic displacement parameters (Å^2^)

**Table:**

	*x*	*y*	*z*	*U*_iso_*/*U*_eq_	
O1	0.9158 (3)	0.3357 (2)	0.61833 (17)	0.0491 (7)	
O2	0.9122 (3)	0.2281 (3)	0.4337 (2)	0.0669 (9)	
H2	0.913 (6)	0.209 (5)	0.494 (4)	0.10 (2)*	
O3	0.3907 (3)	0.1776 (2)	0.49126 (19)	0.0521 (7)	
O4	0.3297 (3)	0.2344 (2)	0.73091 (18)	0.0494 (7)	
O5	0.6836 (3)	0.4867 (3)	0.9172 (2)	0.0599 (8)	
H5	0.680 (5)	0.446 (5)	0.971 (3)	0.081 (18)*	
O6	0.4041 (3)	0.4066 (3)	0.87839 (16)	0.0475 (7)	
O7	0.6399 (3)	0.2797 (3)	0.96522 (19)	0.0672 (9)	
O8	0.5552 (4)	0.3419 (3)	0.3044 (2)	0.0729 (9)	
O9	0.2353 (3)	0.5058 (3)	0.8094 (2)	0.0594 (8)	
O10	−0.0418 (3)	0.2759 (4)	1.1522 (2)	0.0881 (12)	
N1	0.7470 (3)	0.1413 (3)	0.5736 (2)	0.0465 (8)	
C1	0.8099 (4)	0.3994 (4)	0.5738 (2)	0.0451 (9)	
H1	0.8112	0.4757	0.6018	0.054*	
C2	0.8247 (5)	0.4144 (4)	0.4702 (3)	0.0565 (11)	
H2A	0.7606	0.4707	0.4467	0.068*	
H2B	0.9135	0.4431	0.4594	0.068*	
C3	0.8031 (4)	0.3045 (4)	0.4177 (3)	0.0559 (11)	
H3	0.8009	0.3239	0.3512	0.067*	
C4	0.6623 (4)	0.2487 (4)	0.4374 (3)	0.0474 (9)	
C5	0.5815 (4)	0.3212 (3)	0.5059 (2)	0.0418 (8)	
H5A	0.5515	0.3931	0.4769	0.050*	
C6	0.4632 (4)	0.2573 (3)	0.5496 (2)	0.0411 (8)	
H6	0.3997	0.3151	0.5697	0.049*	
C7	0.5253 (4)	0.1991 (3)	0.6387 (2)	0.0403 (8)	
H7	0.5067	0.1169	0.6376	0.048*	
C8	0.4703 (4)	0.2537 (3)	0.7257 (2)	0.0405 (8)	
C9	0.4799 (4)	0.3852 (3)	0.7156 (2)	0.0405 (9)	
H9	0.4018	0.4142	0.6795	0.049*	
C10	0.6107 (4)	0.4228 (3)	0.6692 (2)	0.0372 (8)	
H10	0.5921	0.4964	0.6387	0.045*	
C11	0.6739 (3)	0.3439 (3)	0.5953 (2)	0.0370 (8)	
C12	0.7097 (4)	0.4468 (4)	0.7546 (2)	0.0453 (9)	
H12A	0.7890	0.3998	0.7510	0.054*	
H12B	0.7365	0.5264	0.7556	0.054*	
C13	0.6353 (4)	0.4177 (4)	0.8417 (3)	0.0457 (9)	
C14	0.4927 (4)	0.4450 (3)	0.8096 (2)	0.0425 (9)	
H14	0.4827	0.5276	0.8008	0.051*	
C15	0.5436 (4)	0.2116 (3)	0.8176 (3)	0.0464 (9)	
H15A	0.4759	0.1954	0.8621	0.056*	
H15B	0.5866	0.1397	0.8040	0.056*	
C16	0.6492 (4)	0.2893 (4)	0.8665 (3)	0.0493 (10)	
H16	0.7380	0.2633	0.8498	0.059*	
C17	0.6762 (4)	0.2208 (3)	0.6342 (2)	0.0398 (8)	
H17	0.7180	0.2200	0.6972	0.048*	
C19	0.6822 (5)	0.1301 (4)	0.4802 (3)	0.0539 (11)	
H19A	0.5964	0.0925	0.4845	0.065*	
H19B	0.7374	0.0841	0.4409	0.065*	
C20	0.7676 (5)	0.0285 (4)	0.6163 (3)	0.0635 (13)	
H20A	0.8017	−0.0231	0.5701	0.076*	
H20B	0.6821	−0.0010	0.6345	0.076*	
C21	0.8631 (7)	0.0297 (5)	0.7004 (4)	0.105 (2)	
H21A	0.9388	0.0766	0.6878	0.157*	
H21B	0.8925	−0.0467	0.7141	0.157*	
H21C	0.8187	0.0601	0.7530	0.157*	
C22	1.0347 (4)	0.3991 (5)	0.6345 (3)	0.0698 (13)	
H22A	1.0703	0.4202	0.5759	0.105*	
H22B	1.0990	0.3534	0.6694	0.105*	
H22C	1.0157	0.4668	0.6693	0.105*	
C23	0.5861 (5)	0.2339 (4)	0.3427 (3)	0.0587 (11)	
H23A	0.499 (5)	0.194 (4)	0.343 (3)	0.063 (13)*	
H23B	0.651 (4)	0.192 (4)	0.298 (3)	0.054 (12)*	
C24	0.4958 (8)	0.3360 (7)	0.2134 (4)	0.118 (3)	
H24A	0.5539	0.2953	0.1735	0.177*	
H24B	0.4814	0.4119	0.1897	0.177*	
H24C	0.4118	0.2968	0.2151	0.177*	
C25	0.2723 (5)	0.2212 (5)	0.4519 (4)	0.0827 (16)	
H25A	0.2263	0.1625	0.4165	0.124*	
H25B	0.2918	0.2839	0.4116	0.124*	
H25C	0.2169	0.2474	0.5005	0.124*	
C26	0.2866 (5)	0.1196 (4)	0.7395 (3)	0.0647 (13)	
H26A	0.3629	0.0718	0.7564	0.078*	
H26B	0.2493	0.0935	0.6797	0.078*	
C27	0.1869 (9)	0.1074 (8)	0.8093 (6)	0.152 (4)	
H27A	0.1193	0.1648	0.7996	0.228*	
H27B	0.2287	0.1161	0.8704	0.228*	
H27C	0.1468	0.0331	0.8040	0.228*	
C28	0.2758 (4)	0.4412 (4)	0.8697 (2)	0.0443 (9)	
C29	0.1965 (4)	0.3935 (4)	0.9440 (2)	0.0457 (9)	
C30	0.0640 (5)	0.4273 (5)	0.9512 (3)	0.0725 (15)	
H30	0.0266	0.4791	0.9083	0.087*	
C31	−0.0113 (5)	0.3857 (6)	1.0199 (4)	0.0841 (18)	
H31	−0.1000	0.4083	1.0230	0.101*	
C32	0.0418 (4)	0.3107 (5)	1.0848 (3)	0.0608 (12)	
C33	0.1710 (4)	0.2724 (5)	1.0780 (3)	0.0645 (13)	
H33	0.2063	0.2182	1.1196	0.077*	
C34	0.2476 (4)	0.3163 (4)	1.0078 (3)	0.0577 (11)	
H34	0.3358	0.2926	1.0041	0.069*	
C35	0.0086 (5)	0.1992 (5)	1.2220 (3)	0.0764 (15)	
H35A	0.0423	0.1319	1.1929	0.115*	
H35B	−0.0618	0.1784	1.2619	0.115*	
H35C	0.0794	0.2355	1.2582	0.115*	
C36	0.6919 (7)	0.1752 (5)	1.0015 (4)	0.100 (2)	
H36A	0.7764	0.1597	0.9751	0.149*	
H36B	0.6306	0.1144	0.9859	0.149*	
H36C	0.7038	0.1808	1.0681	0.149*	

### Atomic displacement parameters (Å^2^)

**Table:**

	*U*^11^	*U*^22^	*U*^33^	*U*^12^	*U*^13^	*U*^23^
O1	0.0442 (15)	0.0442 (16)	0.0588 (15)	−0.0045 (13)	0.0015 (12)	0.0027 (14)
O2	0.0565 (19)	0.077 (2)	0.068 (2)	0.0097 (19)	0.0158 (15)	−0.0040 (19)
O3	0.0478 (16)	0.0418 (16)	0.0661 (16)	−0.0040 (13)	−0.0061 (13)	−0.0134 (14)
O4	0.0397 (14)	0.0444 (15)	0.0647 (16)	−0.0053 (13)	0.0112 (12)	0.0001 (14)
O5	0.068 (2)	0.060 (2)	0.0502 (16)	−0.0028 (17)	−0.0112 (14)	−0.0109 (16)
O6	0.0440 (14)	0.0554 (17)	0.0433 (13)	0.0083 (14)	0.0046 (11)	0.0043 (13)
O7	0.086 (2)	0.067 (2)	0.0473 (15)	0.0082 (19)	−0.0039 (15)	0.0120 (15)
O8	0.099 (3)	0.065 (2)	0.0528 (16)	0.001 (2)	−0.0128 (16)	−0.0016 (17)
O9	0.0577 (18)	0.0659 (19)	0.0540 (16)	0.0144 (16)	−0.0042 (13)	0.0077 (15)
O10	0.063 (2)	0.122 (4)	0.081 (2)	0.006 (2)	0.0232 (17)	0.026 (2)
N1	0.054 (2)	0.0347 (18)	0.0518 (18)	0.0082 (16)	0.0081 (15)	−0.0010 (15)
C1	0.045 (2)	0.040 (2)	0.050 (2)	−0.0021 (18)	0.0019 (16)	−0.0039 (18)
C2	0.058 (3)	0.056 (3)	0.056 (2)	−0.004 (2)	0.0084 (19)	0.009 (2)
C3	0.058 (3)	0.063 (3)	0.048 (2)	0.003 (2)	0.0096 (18)	0.002 (2)
C4	0.051 (2)	0.046 (2)	0.0460 (19)	0.000 (2)	0.0048 (16)	−0.0061 (18)
C5	0.049 (2)	0.0318 (19)	0.0441 (18)	−0.0024 (18)	0.0002 (16)	0.0022 (17)
C6	0.0419 (19)	0.0340 (19)	0.0474 (19)	−0.0031 (17)	0.0014 (15)	−0.0055 (17)
C7	0.043 (2)	0.0269 (18)	0.051 (2)	−0.0019 (16)	0.0044 (16)	−0.0024 (16)
C8	0.0381 (19)	0.033 (2)	0.050 (2)	−0.0009 (16)	0.0018 (15)	0.0008 (17)
C9	0.041 (2)	0.036 (2)	0.0446 (19)	0.0041 (17)	0.0035 (15)	−0.0027 (16)
C10	0.0421 (19)	0.0269 (18)	0.0429 (17)	−0.0009 (16)	0.0065 (15)	0.0006 (15)
C11	0.0391 (19)	0.0306 (18)	0.0410 (17)	−0.0024 (16)	−0.0007 (14)	−0.0006 (15)
C12	0.046 (2)	0.041 (2)	0.050 (2)	−0.0084 (18)	0.0030 (16)	−0.0012 (18)
C13	0.046 (2)	0.046 (2)	0.0450 (19)	0.0033 (19)	−0.0018 (16)	−0.0037 (18)
C14	0.050 (2)	0.0351 (19)	0.0427 (19)	0.0018 (18)	0.0045 (16)	−0.0020 (17)
C15	0.049 (2)	0.041 (2)	0.050 (2)	0.0055 (19)	0.0087 (17)	0.0095 (18)
C16	0.049 (2)	0.051 (3)	0.048 (2)	0.005 (2)	−0.0001 (17)	0.0098 (19)
C17	0.043 (2)	0.0332 (19)	0.0431 (18)	0.0003 (17)	0.0022 (15)	−0.0013 (16)
C19	0.060 (3)	0.040 (2)	0.063 (2)	0.003 (2)	0.016 (2)	−0.007 (2)
C20	0.074 (3)	0.041 (2)	0.077 (3)	0.018 (2)	0.018 (2)	0.003 (2)
C21	0.139 (6)	0.073 (4)	0.100 (4)	0.046 (4)	−0.024 (4)	0.020 (3)
C22	0.046 (2)	0.073 (3)	0.091 (3)	−0.017 (3)	0.003 (2)	−0.006 (3)
C23	0.070 (3)	0.053 (3)	0.052 (2)	−0.002 (3)	−0.001 (2)	−0.014 (2)
C24	0.181 (8)	0.097 (5)	0.071 (3)	−0.002 (6)	−0.042 (4)	−0.008 (4)
C25	0.081 (3)	0.051 (3)	0.112 (4)	0.000 (3)	−0.047 (3)	−0.021 (3)
C26	0.064 (3)	0.053 (3)	0.079 (3)	−0.019 (2)	0.016 (2)	−0.002 (2)
C27	0.167 (8)	0.120 (6)	0.175 (7)	−0.090 (6)	0.083 (6)	−0.012 (6)
C28	0.051 (2)	0.044 (2)	0.0372 (18)	0.0057 (19)	−0.0053 (16)	−0.0086 (18)
C29	0.043 (2)	0.053 (2)	0.0401 (17)	0.0025 (19)	−0.0028 (15)	−0.0024 (18)
C30	0.052 (3)	0.094 (4)	0.072 (3)	0.018 (3)	0.004 (2)	0.023 (3)
C31	0.049 (3)	0.112 (5)	0.092 (4)	0.021 (3)	0.014 (2)	0.029 (4)
C32	0.053 (2)	0.074 (3)	0.055 (2)	−0.005 (3)	0.0042 (19)	0.005 (2)
C33	0.053 (3)	0.083 (4)	0.058 (2)	0.007 (3)	0.001 (2)	0.015 (3)
C34	0.045 (2)	0.073 (3)	0.056 (2)	0.012 (2)	0.0083 (18)	0.005 (2)
C35	0.084 (4)	0.080 (4)	0.066 (3)	−0.009 (3)	0.016 (3)	0.005 (3)
C36	0.139 (6)	0.083 (4)	0.074 (3)	0.019 (4)	−0.023 (3)	0.036 (3)

### Geometric parameters (Å, º)

**Table:**

O1—C1	1.425 (5)	C12—C13	1.521 (5)
O1—C22	1.414 (5)	C13—C14	1.516 (5)
O2—H2	0.89 (6)	C13—C16	1.550 (6)
O2—C3	1.424 (5)	C14—H14	0.9800
O3—C6	1.430 (4)	C15—H15A	0.9700
O3—C25	1.388 (6)	C15—H15B	0.9700
O4—C8	1.431 (4)	C15—C16	1.541 (6)
O4—C26	1.419 (5)	C16—H16	0.9800
O5—H5	0.91 (5)	C17—H17	0.9800
O5—C13	1.419 (5)	C19—H19A	0.9700
O6—C14	1.429 (4)	C19—H19B	0.9700
O6—C28	1.348 (5)	C20—H20A	0.9700
O7—C16	1.427 (4)	C20—H20B	0.9700
O7—C36	1.419 (6)	C20—C21	1.507 (7)
O8—C23	1.407 (6)	C21—H21A	0.9600
O8—C24	1.412 (6)	C21—H21B	0.9600
O9—C28	1.206 (5)	C21—H21C	0.9600
O10—C32	1.369 (5)	C22—H22A	0.9600
O10—C35	1.420 (6)	C22—H22B	0.9600
N1—C17	1.475 (5)	C22—H22C	0.9600
N1—C19	1.469 (5)	C23—H23A	0.99 (5)
N1—C20	1.466 (5)	C23—H23B	1.05 (4)
C1—H1	0.9800	C24—H24A	0.9600
C1—C2	1.511 (5)	C24—H24B	0.9600
C1—C11	1.553 (5)	C24—H24C	0.9600
C2—H2A	0.9700	C25—H25A	0.9600
C2—H2B	0.9700	C25—H25B	0.9600
C2—C3	1.502 (6)	C25—H25C	0.9600
C3—H3	0.9800	C26—H26A	0.9700
C3—C4	1.590 (6)	C26—H26B	0.9700
C4—C5	1.553 (5)	C26—C27	1.452 (8)
C4—C19	1.528 (6)	C27—H27A	0.9600
C4—C23	1.539 (6)	C27—H27B	0.9600
C5—H5A	0.9800	C27—H27C	0.9600
C5—C6	1.556 (5)	C28—C29	1.468 (5)
C5—C11	1.571 (5)	C29—C30	1.394 (6)
C6—H6	0.9800	C29—C34	1.369 (6)
C6—C7	1.554 (5)	C30—H30	0.9300
C7—H7	0.9800	C30—C31	1.359 (7)
C7—C8	1.528 (5)	C31—H31	0.9300
C7—C17	1.537 (5)	C31—C32	1.370 (7)
C8—C9	1.549 (5)	C32—C33	1.377 (6)
C8—C15	1.560 (5)	C33—H33	0.9300
C9—H9	0.9800	C33—C34	1.391 (6)
C9—C10	1.559 (5)	C34—H34	0.9300
C9—C14	1.520 (5)	C35—H35A	0.9600
C10—H10	0.9800	C35—H35B	0.9600
C10—C11	1.561 (5)	C35—H35C	0.9600
C10—C12	1.568 (5)	C36—H36A	0.9600
C11—C17	1.545 (5)	C36—H36B	0.9600
C12—H12A	0.9700	C36—H36C	0.9600
C12—H12B	0.9700		
			
C22—O1—C1	113.9 (3)	C16—C15—H15B	107.5
C3—O2—H2	107 (4)	O7—C16—C13	107.2 (3)
C25—O3—C6	113.6 (3)	O7—C16—C15	109.4 (3)
C26—O4—C8	117.3 (3)	O7—C16—H16	108.6
C13—O5—H5	109 (3)	C13—C16—H16	108.6
C28—O6—C14	117.3 (3)	C15—C16—C13	114.5 (3)
C36—O7—C16	113.1 (4)	C15—C16—H16	108.6
C23—O8—C24	113.2 (4)	N1—C17—C7	114.8 (3)
C32—O10—C35	118.4 (4)	N1—C17—C11	112.1 (3)
C19—N1—C17	112.9 (3)	N1—C17—H17	109.9
C20—N1—C17	112.7 (3)	C7—C17—C11	99.8 (3)
C20—N1—C19	110.4 (3)	C7—C17—H17	109.9
O1—C1—H1	107.3	C11—C17—H17	109.9
O1—C1—C2	113.5 (3)	N1—C19—C4	109.3 (3)
O1—C1—C11	109.6 (3)	N1—C19—H19A	109.8
C2—C1—H1	107.3	N1—C19—H19B	109.8
C2—C1—C11	111.7 (3)	C4—C19—H19A	109.8
C11—C1—H1	107.3	C4—C19—H19B	109.8
C1—C2—H2A	109.2	H19A—C19—H19B	108.3
C1—C2—H2B	109.2	N1—C20—H20A	108.9
H2A—C2—H2B	107.9	N1—C20—H20B	108.9
C3—C2—C1	112.1 (4)	N1—C20—C21	113.5 (4)
C3—C2—H2A	109.2	H20A—C20—H20B	107.7
C3—C2—H2B	109.2	C21—C20—H20A	108.9
O2—C3—C2	111.4 (4)	C21—C20—H20B	108.9
O2—C3—H3	106.7	C20—C21—H21A	109.5
O2—C3—C4	113.2 (4)	C20—C21—H21B	109.5
C2—C3—H3	106.7	C20—C21—H21C	109.5
C2—C3—C4	111.8 (3)	H21A—C21—H21B	109.5
C4—C3—H3	106.7	H21A—C21—H21C	109.5
C5—C4—C3	112.3 (3)	H21B—C21—H21C	109.5
C19—C4—C3	110.1 (4)	O1—C22—H22A	109.5
C19—C4—C5	107.7 (3)	O1—C22—H22B	109.5
C19—C4—C23	107.6 (4)	O1—C22—H22C	109.5
C23—C4—C3	107.4 (3)	H22A—C22—H22B	109.5
C23—C4—C5	111.6 (3)	H22A—C22—H22C	109.5
C4—C5—H5A	111.1	H22B—C22—H22C	109.5
C4—C5—C6	114.9 (3)	O8—C23—C4	109.6 (4)
C4—C5—C11	107.7 (3)	O8—C23—H23A	104 (3)
C6—C5—H5A	111.1	O8—C23—H23B	108 (2)
C6—C5—C11	100.6 (3)	C4—C23—H23A	117 (2)
C11—C5—H5A	111.1	C4—C23—H23B	107 (2)
O3—C6—C5	117.0 (3)	H23A—C23—H23B	111 (4)
O3—C6—H6	107.6	O8—C24—H24A	109.5
O3—C6—C7	111.7 (3)	O8—C24—H24B	109.5
C5—C6—H6	107.6	O8—C24—H24C	109.5
C7—C6—C5	104.9 (3)	H24A—C24—H24B	109.5
C7—C6—H6	107.6	H24A—C24—H24C	109.5
C6—C7—H7	110.4	H24B—C24—H24C	109.5
C8—C7—C6	110.1 (3)	O3—C25—H25A	109.5
C8—C7—H7	110.4	O3—C25—H25B	109.5
C8—C7—C17	110.9 (3)	O3—C25—H25C	109.5
C17—C7—C6	104.6 (3)	H25A—C25—H25B	109.5
C17—C7—H7	110.4	H25A—C25—H25C	109.5
O4—C8—C7	111.6 (3)	H25B—C25—H25C	109.5
O4—C8—C9	103.1 (3)	O4—C26—H26A	109.2
O4—C8—C15	109.5 (3)	O4—C26—H26B	109.2
C7—C8—C9	108.2 (3)	O4—C26—C27	112.0 (5)
C7—C8—C15	112.7 (3)	H26A—C26—H26B	107.9
C9—C8—C15	111.4 (3)	C27—C26—H26A	109.2
C8—C9—H9	110.0	C27—C26—H26B	109.2
C8—C9—C10	112.2 (3)	C26—C27—H27A	109.5
C10—C9—H9	110.0	C26—C27—H27B	109.5
C14—C9—C8	112.2 (3)	C26—C27—H27C	109.5
C14—C9—H9	110.0	H27A—C27—H27B	109.5
C14—C9—C10	102.1 (3)	H27A—C27—H27C	109.5
C9—C10—H10	107.0	H27B—C27—H27C	109.5
C9—C10—C11	119.7 (3)	O6—C28—C29	111.2 (3)
C9—C10—C12	103.4 (3)	O9—C28—O6	123.0 (4)
C11—C10—H10	107.0	O9—C28—C29	125.7 (4)
C11—C10—C12	112.0 (3)	C30—C29—C28	119.5 (4)
C12—C10—H10	107.0	C34—C29—C28	122.6 (4)
C1—C11—C5	113.5 (3)	C34—C29—C30	117.9 (4)
C1—C11—C10	105.8 (3)	C29—C30—H30	119.6
C10—C11—C5	114.4 (3)	C31—C30—C29	120.8 (5)
C17—C11—C1	117.6 (3)	C31—C30—H30	119.6
C17—C11—C5	97.8 (3)	C30—C31—H31	119.6
C17—C11—C10	107.9 (3)	C30—C31—C32	120.8 (5)
C10—C12—H12A	110.4	C32—C31—H31	119.6
C10—C12—H12B	110.4	O10—C32—C31	116.0 (4)
H12A—C12—H12B	108.6	O10—C32—C33	124.0 (4)
C13—C12—C10	106.6 (3)	C31—C32—C33	120.0 (4)
C13—C12—H12A	110.4	C32—C33—H33	120.6
C13—C12—H12B	110.4	C32—C33—C34	118.7 (4)
O5—C13—C12	109.6 (3)	C34—C33—H33	120.6
O5—C13—C14	113.4 (3)	C29—C34—C33	121.8 (4)
O5—C13—C16	110.6 (3)	C29—C34—H34	119.1
C12—C13—C16	111.3 (3)	C33—C34—H34	119.1
C14—C13—C12	101.1 (3)	O10—C35—H35A	109.5
C14—C13—C16	110.4 (3)	O10—C35—H35B	109.5
O6—C14—C9	115.9 (3)	O10—C35—H35C	109.5
O6—C14—C13	109.2 (3)	H35A—C35—H35B	109.5
O6—C14—H14	109.7	H35A—C35—H35C	109.5
C9—C14—H14	109.7	H35B—C35—H35C	109.5
C13—C14—C9	102.3 (3)	O7—C36—H36A	109.5
C13—C14—H14	109.7	O7—C36—H36B	109.5
C8—C15—H15A	107.5	O7—C36—H36C	109.5
C8—C15—H15B	107.5	H36A—C36—H36B	109.5
H15A—C15—H15B	107.0	H36A—C36—H36C	109.5
C16—C15—C8	119.1 (3)	H36B—C36—H36C	109.5
C16—C15—H15A	107.5		

### Hydrogen-bond geometry (Å, º)

**Table:**

*D*—H···*A*	*D*—H	H···*A*	*D*···*A*	*D*—H···*A*
O2—H2···O1	0.89 (6)	2.32 (6)	2.932 (4)	126 (5)
O2—H2···N1	0.89 (6)	2.21 (6)	2.845 (4)	128 (5)
O5—H5···O7	0.91 (5)	1.99 (6)	2.562 (5)	120 (4)
C35—H35*B*···O2^i^	0.96	2.56	3.245 (5)	129

Symmetry code: (i) *x*−1, *y*, *z*+1.
